# Differentiating Between Undifferentiated Peripheral Spondyloarthritis From Septic Arthritis: A Case Report

**DOI:** 10.7759/cureus.37742

**Published:** 2023-04-17

**Authors:** Ryuichi Ohta, Chiaki Sano

**Affiliations:** 1 Communiy Care, Unnan City Hospital, Unnan, JPN; 2 Community Medicine Management, Shimane University Faculty of Medicine, Izumo, JPN

**Keywords:** general medicine, rural hospital, older patient, septic arthritis, undifferentiated peripheral spondyloarthritis

## Abstract

Undifferentiated peripheral spondyloarthritis (SpA) and septic arthritis are two distinct differential diagnoses for patients with acute-onset monoarthritis. Effective history-taking and thorough physical examination are essential to differentiate between these two diseases. Precise follow-up can be critical for diagnosing undifferentiated peripheral SpA. Herein, we report our experience with two cases that required differentiation between undifferentiated peripheral SpA and septic arthritis. This case series shows the importance of ruling out septic arthritis promptly and considering the possibility of undifferentiated peripheral PsA based on clinical findings and imaging tests.

## Introduction

Undifferentiated peripheral spondyloarthritis (SpA) and septic arthritis are two distinct differential diagnoses in patients with acute monoarthritis [1]. Peripheral SpA is inflammatory arthritis that affects the joints of the upper and lower limbs and the spine [2, 3]. Among peripheral SpAs, undifferentiated SpA is challenging to diagnose, and various diseases should be differentiated [4]. A critical differential diagnosis of SpA is septic arthritis, a joint infection caused by various organisms, such as the skin colonizer Staphylococcus aureus, with a mortality rate of 7% [5]. Septic arthritis should be ruled out for effective diagnosis of undifferentiated SpA.

Detailed history taking and in-depth physical examinations are essential to differentiate between the two diseases. The duration of joint pain and the risk factors for septic arthritis such as age, immunological conditions, and orthopedic surgeries, should be considered [5]. Ultrasound and magnetic resonance imaging (MRI) is useful for detecting inflamed parts such as the joints' synovium, tendon, and other connective tissues [3].

The prevalence of multiple joint pain and arthritis is high among older patients; hence, primary care physicians should investigate them comprehensively to diagnose undifferentiated peripheral SpA or septic arthritis [6]. Herein, we report our experience differentiating undifferentiated peripheral SpA from septic arthritis. We discuss the main differences between peripheral SpA and septic arthritis, including its cause (inflammatory vs. infectious) and associated symptoms (joint pain and swelling vs. severe joint pain, redness, swelling, and warmth over the affected joint).

## Case presentation

Case 1

A 70-year-old male presented to our hospital with acute onset of pain in the left middle finger while working the previous day. The pain and swelling in the left third finger gradually increased. The patient had no history of other joint pain, chills, fever, or night sweats. He had a past medical history of hypertension, for which he was administered amlodipine (5 mg/day).

**Figure 1:**
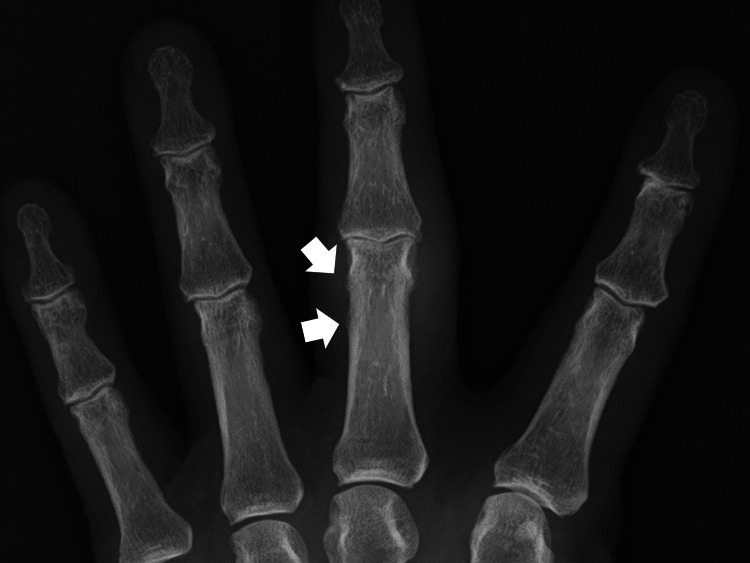
X-ray of the left hand showing osteophytes at the site of tendon attachment around the proximal interphalangeal joint (white arrows).

The vital signs and body temperature were within normal ranges. The patient was well-oriented to time, place, and person. Physical examination revealed swelling and local warmth of the proximal interphalangeal joint of the left middle finger without redness. No other abnormal joint, skin, or nail findings were noted. No obvious abnormalities were observed in the chest or abdomen. Laboratory tests revealed normal levels of inflammatory markers, whereas tests for rheumatoid factor (RF) and anti-citrullinated protein antibodies (ACPA) were negative. A radiograph of the hand showed an osteophyte at the tendon attachment site at the proximal interphalangeal joint (Figure 1).

**Figure 2:**
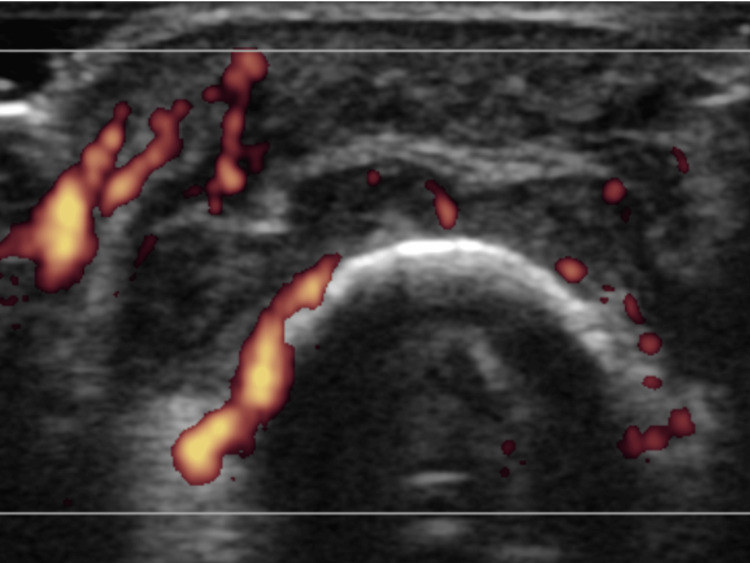
Ultrasound of the interphalangeal joint of the left middle finger showing increased vascular flow at the site of tendon attachment.

Ultrasonography revealed high-intensity vascular flow at the tendon attachment site around the third proximal interphalangeal joint in the left hand (Figure 2).

The patient was initially suspected of having septic arthritis based on these findings. The patient was treated with 200 mg/day of minocycline. The patient’s symptoms did not improve three days later, as confirmed through ultrasonography. Thus, he was clinically diagnosed with undifferentiated peripheral SpA. Diclofenac was administered to alleviate the symptoms. The outpatient laboratory tests did not show elevated uric acid levels at the follow-up. The patient's symptoms were alleviated with Diclofenac daily in the outpatient department.

Case 2

A 73-year-old female presented to our hospital with acute-onset pain in the right middle finger while at work two days prior to the visit. The pain and swelling in the right middle finger gradually increased. The patient had no history of joint pain, chills, fever, or night sweats. She had a past medical history of osteoporosis for which she was on alendronate (50 mg/month).

**Figure 3:**
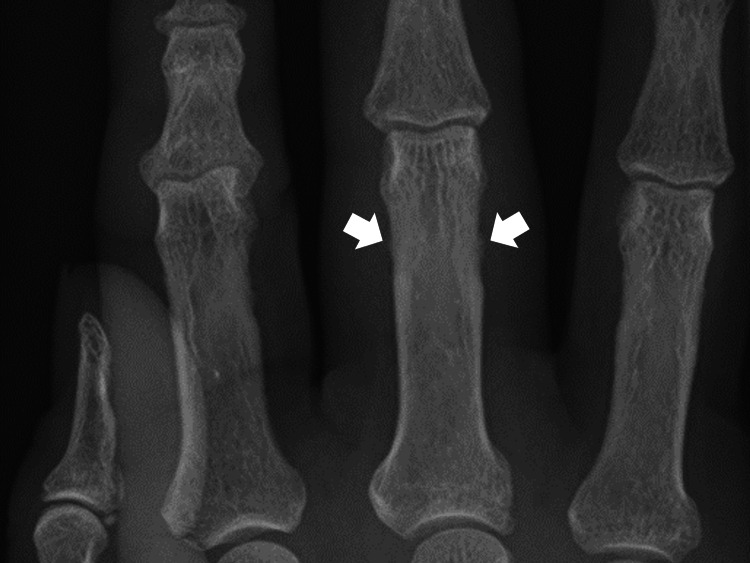
X-ray of the hand showing osteophytes at the site of tendon attachment around the proximal interphalangeal joint of the right middle finger (white arrows).

The vital signs and body temperature were within normal ranges. The patient was well-oriented to time, place, and person. Physical examination revealed swelling and local warmth of the proximal interphalangeal joint of the right middle finger without redness. No other abnormal joint, skin, or nail findings were noted. No obvious abnormalities were observed in the chest or abdomen. Laboratory tests revealed normal levels of inflammatory markers, and tests for RF and ACPA were negative. Radiography of the hand showed changes at the sites of tendon attachment around the proximal interphalangeal joint of the right middle finger (Figure 3).

**Figure 4:**
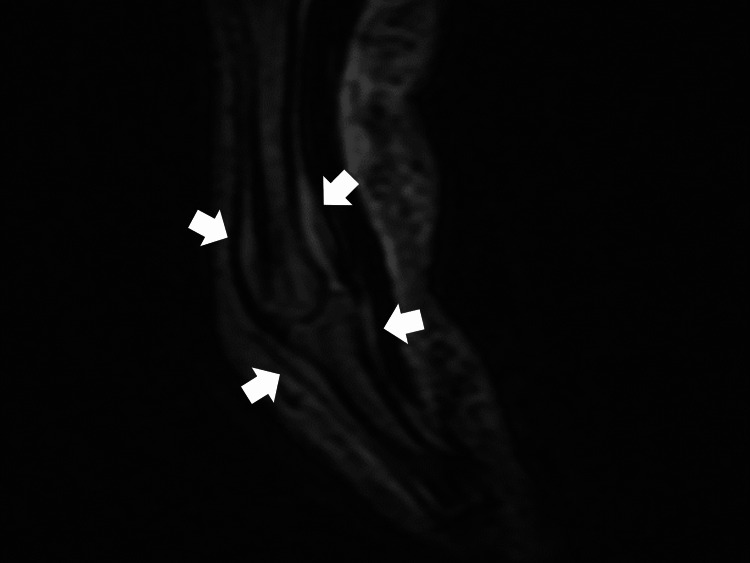
Magnetic resonance imaging of the right hand (short Tau inversion recovery views) showing high-intensity signal areas around the joint, such as at the tendon attachments (white arrows).

Magnetic resonance imaging (MRI) of the right hand showed high-intensity signal areas around the joint, such as at the tendon attachments (Figure 4).

Based on these findings, the patient was initially diagnosed with cellulitis, for which 3g/day of cephalexin was initiated. The patient’s symptoms did not improve significantly one week later. Thus, she was clinically diagnosed with undifferentiated peripheral SpA. Diclofenac was administered to alleviate the symptoms. The outpatient laboratory tests did not show elevated uric acid levels at the follow-up. The patient's symptoms were alleviated with diclofenac daily in the outpatient department.

## Discussion

This case series shows the importance of ruling out septic arthritis promptly, in addition to considering various other possibilities of arthritis, including undifferentiated peripheral PsA, based on the patient’s history. The risk of septic arthritis should first be assessed in patients with acute monoarthritis [5]. Aging, trauma, immunosuppression, and travel history are essential for assessing the risk of septic arthritis. Disease and medication history such as diabetes and autoimmune diseases with immunosuppressant use should be adequately checked [7]. Vital signs indicate an improvement in the severity of the condition, particularly in patients with septic arthritis. For a definitive diagnosis, synovial fluid should be analyzed; white blood cell counts >50,000/indicate a high probability of septic arthritis [5]. However, obtaining synovial fluid from some patients may be difficult. In this case series, synovial fluid was not detected in radiographic imaging. Such patients should be treated for septic arthritis based on comprehensive clinical findings [8]. Blood culture is mandatory in patients with septic arthritis [5]. Positive results should prompt further investigation to identify complications such as infectious endocarditis and multiple systemic abscesses. Most cases of septic arthritis are caused by Staphylococcus, which can cause endocarditis and systemic abscesses [9].

Undifferentiated peripheral SpA is inflammatory arthritis that does not involve specific autoimmune antibodies. The collaboration of primary care physicians and rheumatologists typically manages it with nonsteroidal anti-inflammatory drugs (NSAIDs), disease-modifying antirheumatic drugs (DMARDs), and biological agents [10]. Physical therapy and lifestyle changes are also helpful in managing stiffness and preventing joint destruction. Early diagnosis and treatment of undifferentiated peripheral SpA can prevent long-term damage to the joints and improve quality of life.

Diagnosing undifferentiated peripheral SpA in a primary care setting is challenging because the initial symptoms vary and are similar to other types of arthritis such as rheumatoid arthritis or osteoarthritis [7, 11]. Primary care physicians should focus on the patient's medical history, physical examination, and comprehensive laboratory tests to effectively diagnose undifferentiated peripheral SpA [12]. X-ray or MRI should be performed to detect inflammation of the bones and lesions in the tendons and ligaments [13].

Delayed diagnosis can be a critical issue in primary care. The possibility of undifferentiated peripheral SpA should always be considered in patients presenting with acute-onset joint symptoms of unknown origin [3]. Patients should be referred to a rheumatologist to ensure an effective early diagnosis. Early diagnosis and treatment of PsA can prevent long-term joint damage and improve the patient's quality of life [14]. However, the lack of rheumatologists in rural regions can deter effective diagnosis. In addition, the musculoskeletal and rheumatic symptoms in older patients may vary, causing difficulty in making a diagnosis [15, 16]. Thus, general physicians in rural regions should be aware of and consider undifferentiated peripheral SpA in older patients with acute-onset monoarthritis after septic arthritis is ruled out.

## Conclusions

This case series shows the importance of ruling out septic arthritis promptly in addition to considering other inflammatory joint diseases. Delays in diagnosis are a critical issue in primary care. In a primary care setting, the possibility of undifferentiated peripheral SpA should be considered in patients with acute-onset joint symptoms of unknown etiology.
